# Transcriptomic responses of skin to different micro-injury modalities: fire filiform needling, ablative fractional CO_2_ laser, and filiform needling

**DOI:** 10.1007/s10103-026-05004-1

**Published:** 2026-08-25

**Authors:** Xixia Dai, Faisal Amin, Jianjian Zhu, Haoran Guo, Zhen Tang, Yushan Zhang, Jing Chen, Qinghai Zeng, Li Lei

**Affiliations:** 1 Department of Dermatology, Third Xiangya Hospital, Central South University, Changsha, 410013 Hunan China; 2 Department of Dermatology, Changde Hospital, Xiangya School of Medicine, Central South University (The First People’s Hospital of Changde city), Changde, 415000 China

**Keywords:** Micro-injury, Ablative fractional CO_2_ laser, Fire filiform needling, Filiform needling, Skin repair

## Abstract

Repair strategies predicated on controlled micro-injury are widely applied in dermatology; however, the distinct molecular cascades initiated by different injury modalities remain incompletely characterized. To systematically delineate and compare the acute transcriptomic landscapes of mouse skin following fire filiform needling (FFN), ablative fractional CO_2_ laser (AFCO_2_L), and conventional filiform needling (FN), we performed high-throughput RNA sequencing on dorsal skin samples harvested from C57BL/6J mice one day after treatment with FFN (combining thermal and mechanical stimuli), AFCO_2_L (purely thermal), or FN (purely mechanical). All three interventions disrupted skin barrier integrity and activated canonical injury and repair transcriptional programs. Notably, the two modalities involving thermal stimulation (FFN and AFCO_2_L) elicited a more robust and expansive transcriptomic response than purely mechanical FN, including immune activation, cell proliferation, differentiation, cell migration, metabolic reprogramming, and neurovascular remodeling. Although FFN and AFCO_2_L exhibited broadly convergent transcriptional signatures, key distinctions emerged: FFN was preferentially associated with tissue remodeling, vascular processes, and neural regulation, whereas AFCO_2_L displayed stronger enrichment for immune activation and cell migration. This study provides a comprehensive transcriptomic atlas of acute skin responses to distinct micro-injury modalities and underscores the potent role of thermal stimulation in amplifying dermal repair and remodeling. Our findings establish a systematic framework for understanding the mechanistic underpinnings of micro-injury therapies and for refining their clinical application.

**Supplementary Information:**

The online version contains supplementary material available at https://doi.org/10.1007/s10103-026-05004-1.

## Introduction

The skin functions as a vital barrier organ whose homeostasis relies on the coordinated interplay among keratinocytes, fibroblasts, melanocytes, immune cells, nerve endings, and cutaneous appendages. Upon injury, the skin rapidly initiates a tightly orchestrated repair program—encompassing cell proliferation, inflammation, metabolic adaptation, and ECM remodeling—to restore barrier integrity [1].

In recent years, controlled micro-injury has emerged as an effective strategy to harness endogenous repair mechanisms for therapeutic benefit in dermatology and regenerative medicine. Microneedling, which generates microchannels spanning the epidermis and superficial dermis, facilitates transdermal drug delivery while concurrently activating intrinsic wound-healing cascades. This approach has demonstrated efficacy in conditions such as vitiligo, melasma, acne, and scarring [2–4]. Filiform needling (FN), which entails the insertion of a slender acupuncture needle into the dermis, constitutes a form of mechanical microdamage analogous to microneedling. In contrast, micro-injury modalities that incorporate a thermal component can further modulate the intensity of inflammation and the depth of tissue remodeling. Ablative fractional CO_2_ laser (AFCO_2_L), a prototypical purely thermal micro-injury technique, creates an array of microscopic thermal injury zones that induce localized heat stress while sparing intervening healthy tissue, thereby promoting coordinated skin repair [5]. Transcriptomic studies have revealed that such interventions trigger concerted biological responses encompassing inflammation, epidermal regeneration, dermal remodeling, barrier restoration, and immune activation [6–8]. Fire filiform needling (FFN), a traditional therapeutic modality that uniquely couples transient high-temperature stimulation with mechanical penetration, has been investigated in clinical studies as an adjunctive or alternative intervention for several dermatological conditions, including vitiligo, acne, psoriasis, chronic eczema, and neurodermatitis [9–12].

Despite accumulating evidence supporting the therapeutic utility of micro-injury approaches, systematic comparative transcriptomic analyses across distinct modalities remain limited. To address this knowledge gap, we performed high-throughput RNA sequencing to compare the acute transcriptomic effects of FFN (thermal and mechanical), AFCO_2_L (thermal), and FN (mechanical) on normal mouse skin. Multi-dimensional analyses were conducted across key biological domains—cell fate regulation, chemotaxis and migration, metabolism, immune responses, ECM remodeling, and neural regulation—to provide mechanistic insights and inform the optimization of micro-injury-based dermatological therapies. Additionally, for select findings related to thermal stimulation, we referenced a previously generated spatial transcriptomics dataset from a vitiligo mouse model subjected to mild heat stress as a supplementary line of evidence.

**Figure:**
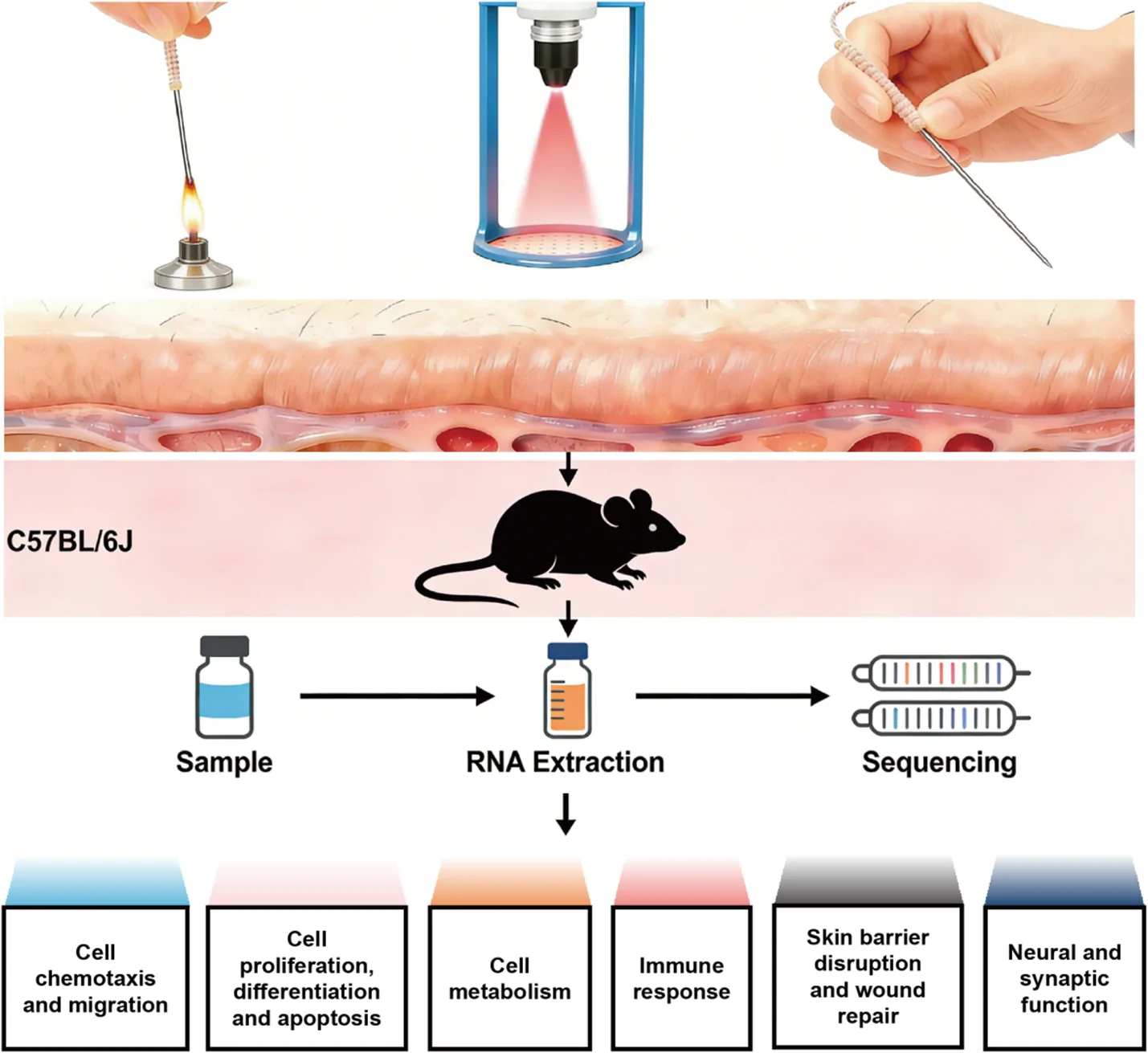

## Materials and methods

### Animal treatment and RNA sequencing

Seven-week-old male C57BL/6J mice were obtained from Hunan SJA Laboratory Animal Co., Ltd. (Hunan, China). All animals were housed under specific pathogen-free (SPF) conditions. Following a 3-day acclimatization period, mice were randomly assigned to experimental groups using a computer-generated randomization scheme.

One day prior to treatment, dorsal hair was removed using electric clippers followed by depilatory cream to create a uniform hairless area of approximately 2 × 2 cm^2^. All interventions were performed under intraperitoneal anesthesia with tribromoethanol. Mice were divided into four groups (*n* = 3 per group): normal control (NC), FFN, AFCO_2_L, and FN. Based on preliminary optimization experiments, the AFCO_2_L group received a single pass of fractional CO_2_ laser treatment (Kinglaser KL type, JiLin, China) on the depilated area using the following parameters: energy 50 mJ, treatment area 2 × 2 cm², and spot spacing 0.6 mm. These parameters were selected to achieve a penetration depth comparable to that of FFN and FN, thereby enhancing cross-modality comparability. For the FFN group, a 0.3 mm diameter stainless steel needle was heated over an alcohol flame until the tip glowed red, then rapidly inserted vertically into the skin to a depth of approximately 2 mm. Punctures were evenly distributed across the treatment field at intervals of 1–3 mm, with the needle withdrawn immediately after each insertion. For the FN group, an identical needle was used to puncture the skin vertically to a depth of approximately 2 mm in a comparable spatial distribution, but without thermal stimulation.

The NC group underwent hair removal and anesthesia only, without any needle or laser intervention.

At 24 h post-treatment, mice were euthanized, and skin samples from the treated area were harvested. Tissues were immediately rinsed with sterile phosphate-buffered saline (PBS), transferred into RNase-free tubes, and snap-frozen in liquid nitrogen. Samples were subsequently pulverized under liquid nitrogen, and total RNA was extracted using TRIzol reagent. High-throughput RNA sequencing was performed on an Illumina platform.

### RNA-seq data analysis

All bioinformatics analyses were conducted using R software (version 4.3.0). Expression matrices were preprocessed and normalized using the tidyverse and reshape2 packages. Genes with extremely low expression were filtered out. Principal component analysis (PCA) was employed to assess global transcriptomic variation and data quality.

Differentially expressed genes (DEGs) were identified using the DESeq2 package, applying thresholds of |log_2_ fold change (log_2_FC)| > 1 and *P* < 0.05. DEGs were visualized using volcano plots and heatmaps. Functional enrichment analysis, including Gene Ontology (GO) and Kyoto Encyclopedia of Genes and Genomes (KEGG) pathway analyses, was performed using the clusterProfiler package. For Gene Set Enrichment Analysis (GSEA), all genes were ranked by log_2_FC, and both GSEA-GO and GSEA-KEGG analyses were executed.

### Spatial transcriptomics analysis

To further explore transcriptional responses associated with sustained thermal stimulation, we analyzed an unpublished spatial transcriptomics dataset generated by our group. In this model, vitiligo-like lesions were induced on the dorsal skin of C57BL/6J mice by topical application of monobenzone cream for 6 weeks. Subsequently, a localized mild thermal stimulus (41 °C heating patch) was applied to the lesional area for 5 weeks. Skin tissues were then collected for spatial transcriptomic sequencing. Following quality control, differential expression analysis and GSEA were performed. These data were utilized to support and contextualize the thermal-related mechanisms observed following FFN and AFCO_2_L treatment.

## Results

### Global transcriptomic features and differential gene expression following FFN, AFCO_2_L, and FN treatment

**Fig. 1:**
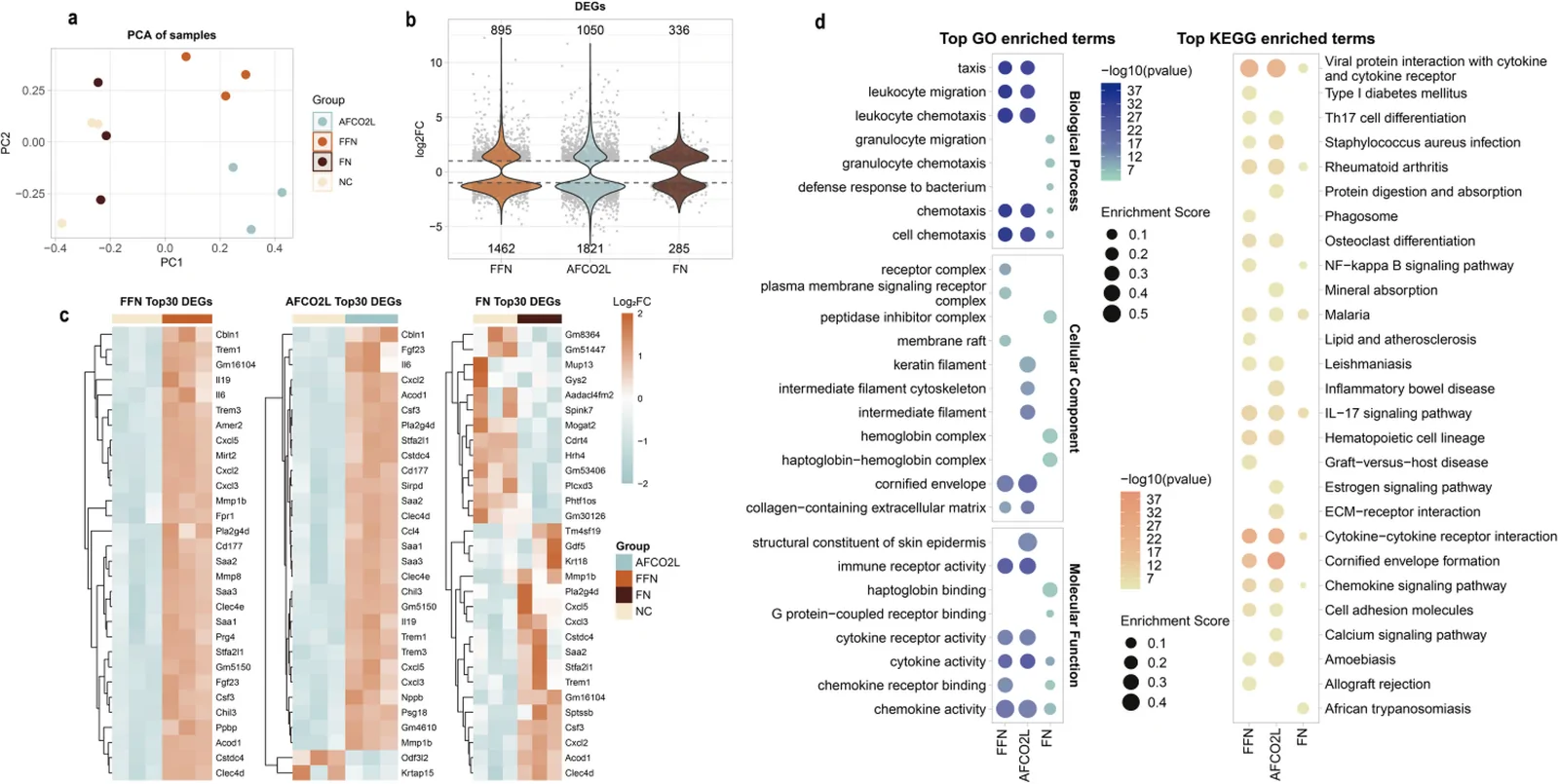
Transcriptomic characteristics and differential gene analysis of mouse skin at day 1 after FFN, AFCO_2_L, and FN treatment. (**a**) PCA depicting the global transcriptomic distribution of all samples. (**b**) Volcano plots illustrating DEGs identified in FN, FFN, and AFCO_2_L groups relative to NC. (**c**) Heatmap displaying the top DEGs ranked by P value in each treatment group compared with NC. (**d**) Bubble plots showing the top enriched GO terms and KEGG pathways ranked by P value. GO results are presented across biological process (BP), cellular component (CC), and molecular function (MF) categories

To characterize the early transcriptomic responses elicited by different micro-injury modalities, we first examined global gene expression profiles in mouse skin at day 1 post-treatment. Gene expression distributions across all samples were comparable after log₂ transformation, with high intra-group consistency and no evident outliers or systematic bias (Fig. S1a). When comparing overall expression patterns, all three treatment groups exhibited varying degrees of deviation from the normal control (NC), with FFN and AFCO_2_L displaying more similar global shifts, whereas FN showed relatively modest changes (Fig. S1b). PCA further demonstrated that FFN and AFCO_2_L samples clustered closely together and clearly segregated from the FN group (Fig. 1a), indicating that thermal stimulation drives a more convergent early transcriptional response.

Differential expression analysis corroborated this trend. Relative to the NC group, we identified 2,357 DEGs (895 upregulated, 1,462 downregulated) in the FFN group, 2,871 DEGs (1,050 upregulated, 1,821 downregulated) in the AFCO_2_L group, and 621 DEGs (336 upregulated, 285 downregulated) in the FN group (Fig. 1b). The substantially higher number of DEGs in the FFN and AFCO_2_L groups underscores that modalities involving thermal stress provoke a more pronounced transcriptomic perturbation than purely mechanical stimulation. A heatmap of the top 30 DEGs (ranked by P value) revealed both shared and treatment-specific transcriptional responses. Genes commonly upregulated across all groups included Cxcl2, Cxcl3, Cxcl5, and Pla2g4d, reflecting a conserved early inflammatory signaling cascade following micro-injury (Fig. 1c).

Functional enrichment analysis further delineated the similarities and differences among the three interventions. FFN and AFCO₂L exhibited highly concordant enrichment profiles in both GO and KEGG analyses, whereas FN displayed a markedly attenuated enrichment landscape, with fewer significantly enriched terms and less pronounced enrichment scores (Fig. 1d). At the biological process level, FFN and AFCO_2_L were prominently enriched in terms related to cell chemotaxis and leukocyte migration, whereas FN was primarily enriched in granulocyte chemotaxis and defense response to bacterium. This suggests that while all treatments rapidly initiate innate immune recruitment, the magnitude of this response differs substantially. In the cellular component category, DEGs in the FFN and AFCO_2_L groups were significantly enriched in structural terms such as collagen-containing extracellular matrix, cornified envelope, intermediate filament cytoskeleton, and keratin filament, indicative of early-stage remodeling of both the epidermal barrier and dermal matrix. At the molecular function level, FFN showed strong enrichment for chemokine activity, cytokine activity, cytokine receptor activity, and immune receptor activity. AFCO_2_L exhibited a similar pattern, with additional enrichment for structural constituent of skin epidermis. FN was primarily enriched for chemokine receptor binding, G protein-coupled receptor binding, and haptoglobin binding, indicating that although all treatments activated intercellular signaling, FFN and AFCO_2_L more robustly engaged immune receptor-mediated pathways. KEGG pathway analysis further supported these observations: FFN and AFCO_2_L were significantly enriched in canonical inflammatory and tissue remodeling pathways, including cytokine-cytokine receptor interaction, chemokine signaling, IL-17 signaling, NF-κB signaling, and ECM-receptor interaction, as well as multiple pathways associated with infection and immune responses. In contrast, enrichment in these pathways was markedly weaker in the FN group.

### Effects of FFN, AFCO_2_L, and FN on cell proliferation, differentiation, and apoptosis

To further elucidate the regulatory influence of distinct micro-injury modalities on skin cell fate, we performed GSEA using all genes ranked by differential expression relative to the NC group. GO terms associated with cell proliferation, differentiation, and apoptosis were selected for presentation.

**Fig. 2:**
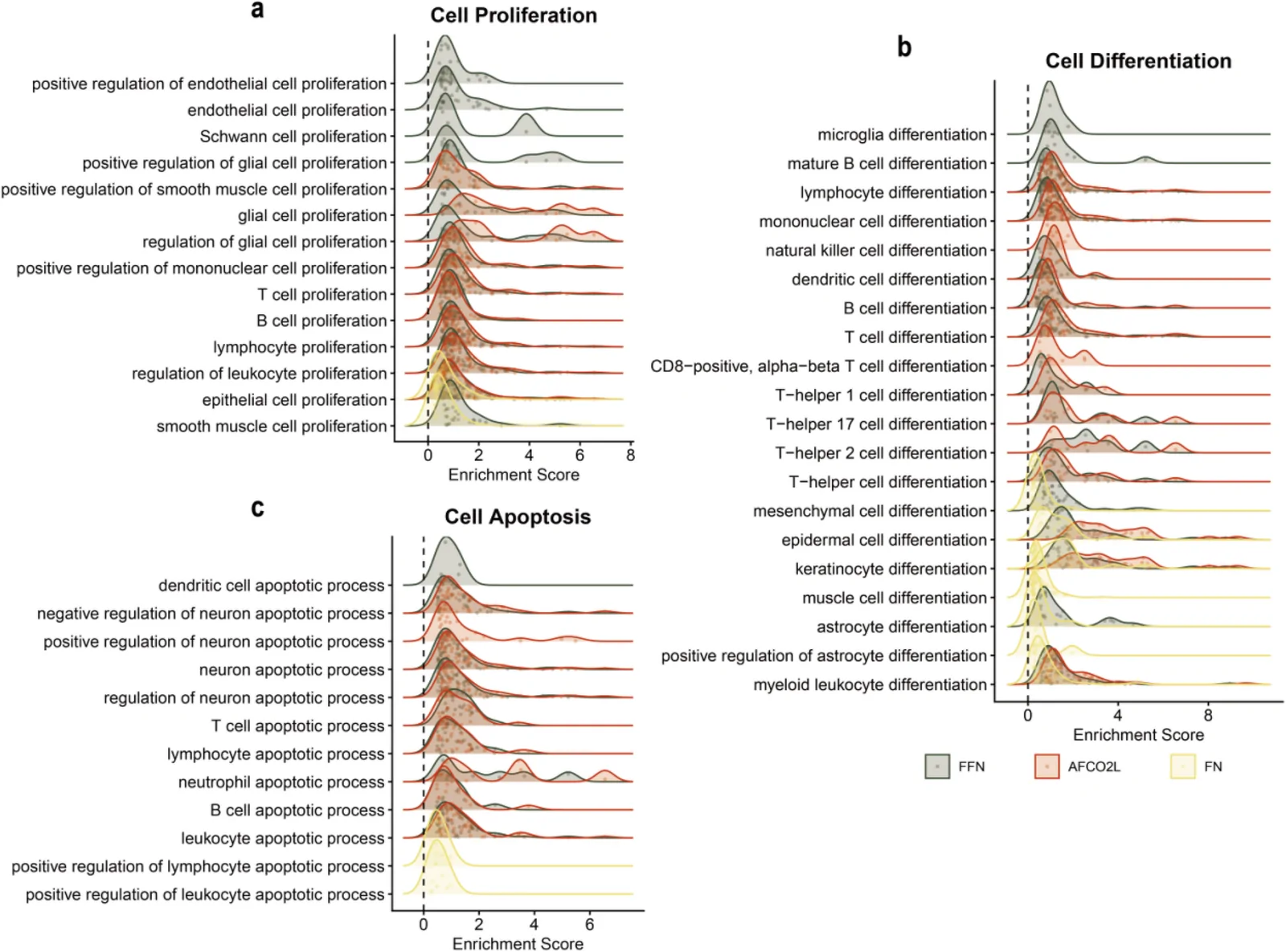
Effects of FFN, AFCO_2_L, and FN on cell proliferation, differentiation, and apoptosis in skin. (**a**–**c**) Ridge plots depicting GSEA_GO enrichment results for cell proliferation (**a**), differentiation (**b**), and apoptosis (**c**) in FFN, AFCO_2_L, and FN groups compared with NC

Regarding cell proliferation, both FFN and AFCO_2_L activated a broad array of proliferative programs linked to skin repair. These included structural cell populations such as epithelial cells and smooth muscle cells, as well as enhanced proliferation signals in immune cells (mononuclear cells and lymphocytes) and neural-related cells (glial cells). This pattern indicates that the inclusion of a thermal stimulus rapidly initiates coordinated cell renewal processes encompassing barrier repair, immune activation, and neuroinflammatory responses. Notably, FFN exhibited additional enrichment for endothelial cell and Schwann cell proliferation, suggesting a more prominent role in vascular remodeling and peripheral nerve-associated cellular renewal during the acute phase. In comparison, FN primarily induced proliferation in epithelial and smooth muscle cells, with lower overall intensity (Fig. 2a).

With respect to cell differentiation, FFN and AFCO_2_L similarly promoted differentiation of skin structural cells—particularly keratinocytes and epidermal cells—while also broadly engaging immune cell differentiation programs, including myeloid leukocytes, mononuclear cells, dendritic cells, T cells, and B cells. These findings suggest that FFN and AFCO₂L promote coordinated differentiation across multiple cell lineages during the acute phase of skin repair. Subtle distinctions were observed between FFN and AFCO_2_L: FFN additionally promoted differentiation of mesenchymal cells, astrocytes, and microglia, whereas AFCO_2_L exhibited stronger enrichment for differentiation of CD8-positive alpha-beta T cells and natural killer cells. FN also induced differentiation across several cell types, including epidermal cells, keratinocytes, mesenchymal cells, muscle cells, myeloid leukocytes, and astrocytes, though overall enrichment was less pronounced (Fig. 2b).

In terms of apoptosis, both FFN and AFCO_2_L significantly activated apoptosis-related transcriptional programs in immune cells, particularly leukocytes and lymphocytes, and concurrently engaged neuronal apoptosis along with its positive and negative regulatory processes. This suggests that, amidst concurrent inflammation and tissue renewal, these modalities initiate fine-tuned regulation of immune and neural cell homeostasis. In contrast, FN primarily enriched for positive regulation of leukocyte and lymphocyte apoptosis, reflecting a more circumscribed apoptotic response (Fig. 2c).

### Effects of FFN, AFCO_2_L, and FN on cell chemotaxis and migration

**Fig. 3:**
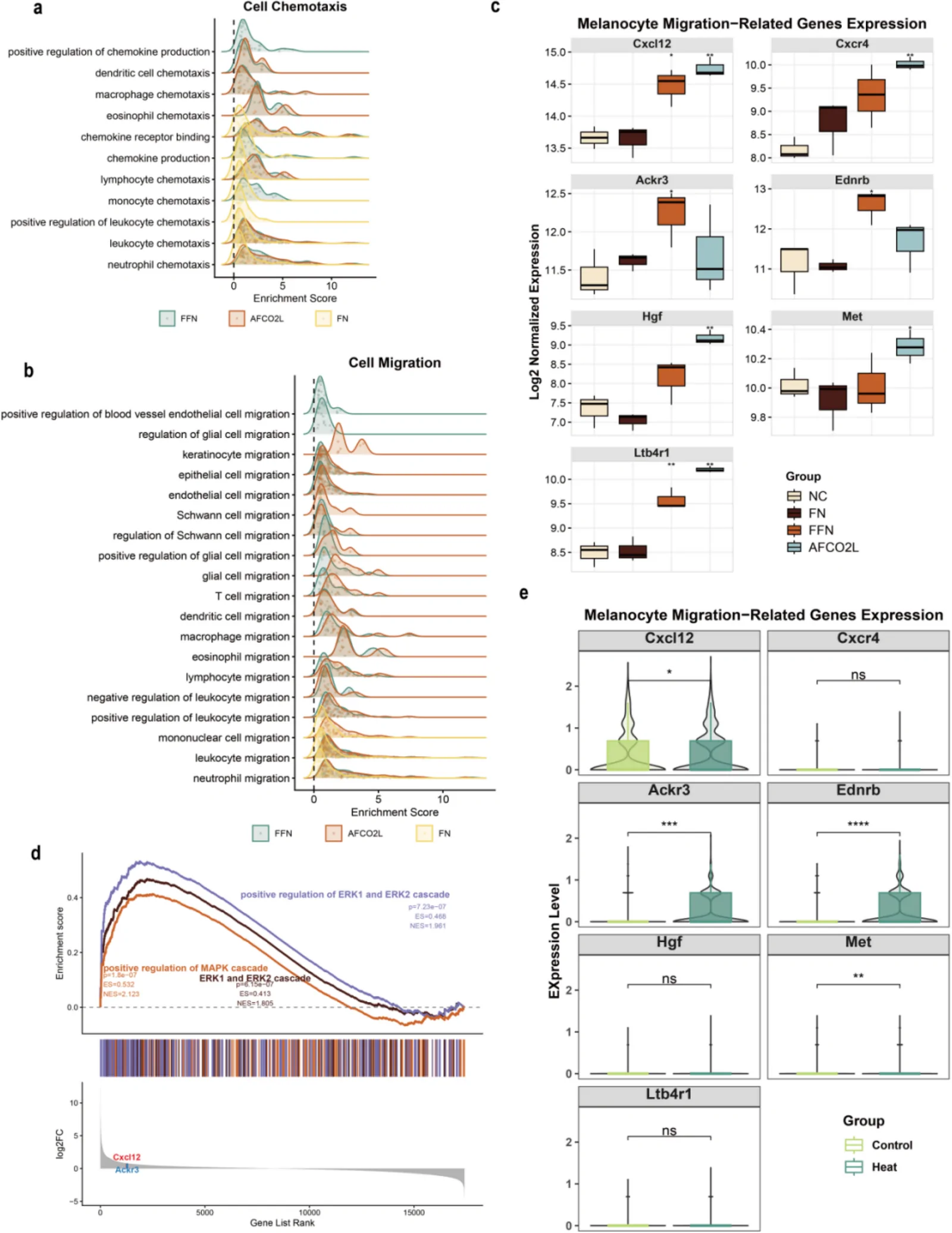
Effects of FFN, AFCO₂L, and FN on cell chemotaxis and migration in skin. (**a**–**b**) Ridge plots showing GSEA_GO enrichment results for cell chemotaxis (**a**) and cell migration (**b**) in FFN, AFCO₂L, and FN groups compared with NC. (**c**) Box plots depicting expression levels of melanocyte migration-related genes across groups. Differences among groups were assessed using one-way ANOVA followed by Tukey’s post-hoc test (*P < 0.05, **P < 0.01). (**d**) GSEA enrichment plot illustrating co-enrichment of Cxcl12 and Ackr3 in ERK1/2 cascade-related terms in the FFN group. (**e**) Box plots showing expression of melanocyte migration–related genes in control and heat groups based on spatial transcriptomics data from a vitiligo mouse model. Statistical significance was determined using Student’s t-test (*P < 0.05, **P < 0.01)

To evaluate the impact of different micro-injury modalities on skin cell chemotaxis and migration, relevant GO terms were extracted from GSEA results for presentation. For chemotaxis-related processes, both FFN and AFCO_2_L groups exhibited significant enrichment across multiple immune cell chemotactic pathways, including leukocyte, neutrophil, lymphocyte, eosinophil, macrophage, and dendritic cell chemotaxis. The FN group was also enriched in chemotaxis terms involving leukocytes, neutrophils, mononuclear cells, and lymphocytes, albeit with lower overall enrichment intensity (Fig. 3a). For migration-related processes, FFN and AFCO_2_L displayed broader activation of migratory programs spanning diverse cell types. Beyond immune cell migration, these included epithelial cells, endothelial cells, and neural-related cells such as glial cells and Schwann cells, suggesting that these two interventions can rapidly engage migration programs associated with tissue repair and neurostructural remodeling. Notably, the FFN group also exhibited enrichment for positive regulation of blood vessel endothelial cell migration, indicating stronger transcriptional activation of vascular-associated migratory processes. In comparison, the FN group primarily promoted migration of immune cells, including leukocytes, neutrophils, and mononuclear cells (Fig. 3b).

Beyond general cell migration, we specifically examined pathways relevant to melanocyte motility, given its critical role in vitiligo repigmentation. Current GSEA gene sets lack a specific term for melanocyte migration. We therefore examined the expression of key genes implicated in melanocyte migration and its regulatory microenvironment. Relative to the NC group, FFN and AFCO_2_L elicited more pronounced transcriptional changes in multiple genes associated with melanocyte migration and chemotaxis, whereas changes in the FN group were comparatively limited (Fig. S2a). The chemokine Cxcl12 was significantly upregulated in both FFN and AFCO_2_L groups, accompanied by differential expression of its cognate receptors Ackr3 and Cxcr4: FFN primarily upregulated Ackr3, whereas AFCO_2_L preferentially upregulated Cxcr4. Additionally, each modality was associated with distinct alterations in other melanocyte migration-related genes—FFN specifically upregulated Ednrb, while AFCO_2_L upregulated Hgf and its receptor Met (Fig. 3c). These findings suggest that FFN and AFCO_2_L may regulate melanocyte migration through the activation of multiple chemokine–receptor signaling axes and growth factor pathways. Further analysis of downstream signaling revealed that, in the GSEA comparison of FFN versus NC, Cxcl12 and Ackr3 were co-enriched in the core gene sets of the ERK1/2 cascade and its positive regulation (Fig. 3d). Similarly, in the AFCO_2_L versus NC comparison, Cxcl12 and Cxcr4 were co-enriched in ERK/MAPK-related pathways (Fig. S2b). This indicates that both FFN and AFCO_2_L may converge on ERK signaling activation via the Cxcl12-receptor axis, albeit through distinct receptor engagement.

Validation in a vitiligo mouse model subjected to mild heat stress further demonstrated that thermal stimulation significantly upregulated the transcriptional levels of Cxcl12, Ackr3, Ednrb, and Met compared with controls (Fig. 3e), lending additional support to a potential role for thermal stimulation in promoting melanocyte migration.

### Effects of FFN, AFCO_2_L, and FN on skin metabolism

**Fig. 4:**
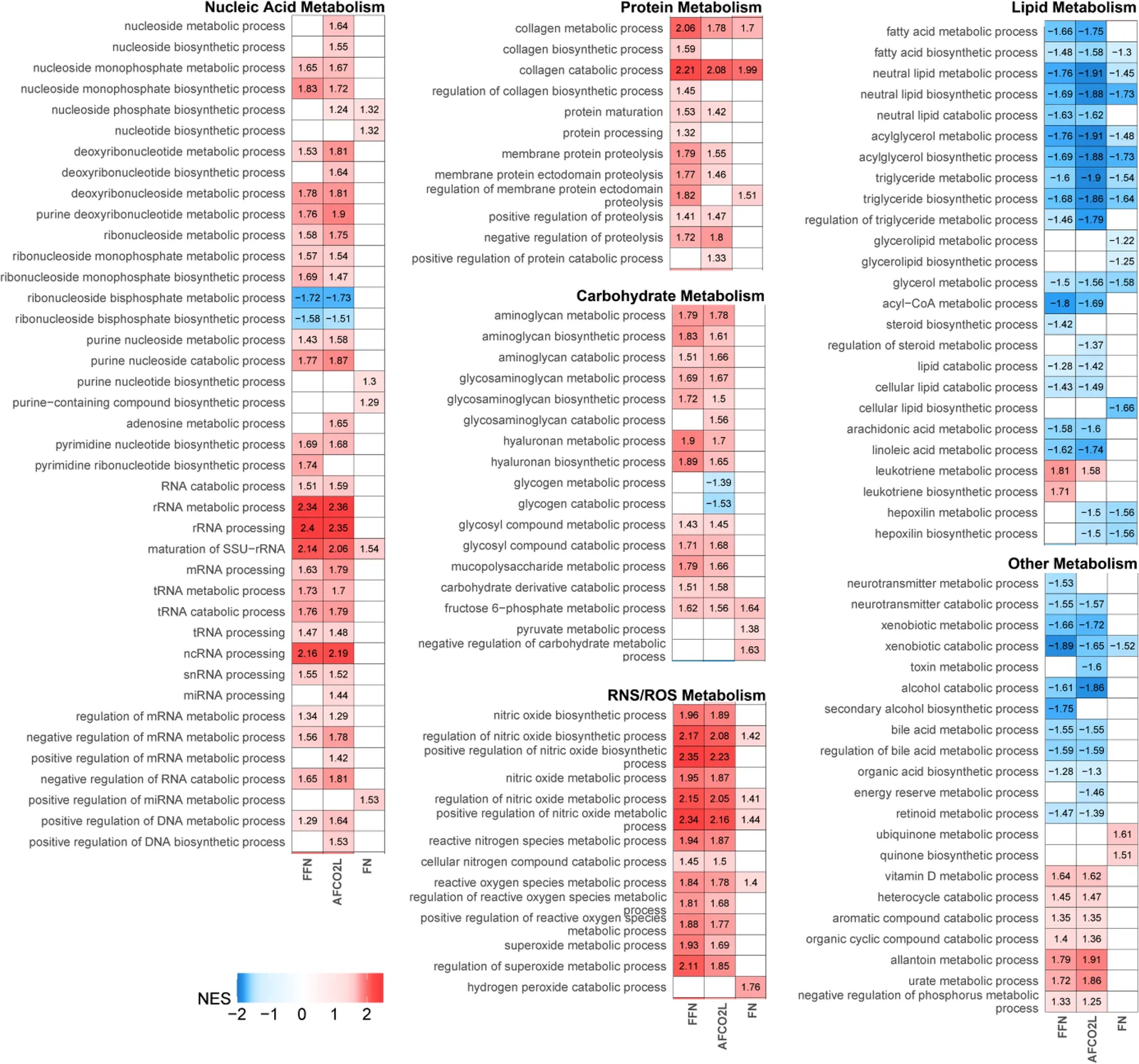
Effects of FFN, AFCO_2_L, and FN on metabolic functions in skin. Heatmap displaying metabolism-related terms from GSEA_GO enrichment results in FFN, AFCO_2_L, and FN groups compared with NC

To systematically evaluate the metabolic consequences of different micro-injury modalities, metabolism-related GO terms were integrated and analyzed based on GSEA_GO results (Fig. 4). FFN and AFCO_2_L exhibited highly consistent regulatory patterns across multiple metabolic modules. At the nucleic acid level, both groups were significantly enriched in nucleoside monophosphate, purine, and pyrimidine nucleotide biosynthetic and metabolic processes, accompanied by enhanced processing and metabolism of rRNA, mRNA, tRNA, ncRNA, and snRNA. With respect to protein metabolism, FFN and AFCO_2_L coordinately activated collagen metabolic and catabolic processes, alongside enrichment in membrane protein proteolysis, protein maturation, and regulation of proteolysis. Carbohydrate metabolism was also concurrently activated, encompassing glycosaminoglycan, hyaluronan, and fructose-6-phosphate metabolic processes. In oxidative and nitrogen-related modules, both groups exhibited activation of nitric oxide (NO) biosynthetic and metabolic processes, as well as reactive oxygen species (ROS) and reactive nitrogen species metabolic processes.

In contrast to these activated modules, lipid-related metabolic pathways were consistently downregulated in both FFN and AFCO_2_L groups, including fatty acid, triglyceride, cholesterol, sphingolipid, and arachidonic acid metabolic processes. Notably, leukotriene biosynthetic and metabolic processes were upregulated, suggesting selective activation of inflammatory lipid mediators amidst a broader suppression of lipid metabolism. Beyond the core metabolic domains, FFN and AFCO_2_L also demonstrated highly concordant regulation of additional pathways, including upregulation of allantoin, urate, and vitamin D metabolic processes, and enhanced catabolism of heterocycles and aromatic compounds. Conversely, retinoid metabolism, bile acid metabolism, xenobiotic metabolic and catabolic processes, alcohol catabolic processes, and neurotransmitter metabolic and catabolic processes were downregulated.

In comparison, the FN group also engaged multiple metabolic modules, primarily characterized by upregulation of nucleotide biosynthesis, rRNA maturation, collagen metabolism, and NO/ROS-related processes, alongside downregulation of lipid-related processes such as neutral lipid, triglyceride, and fatty acid biosynthesis. However, both the number of enriched terms and the magnitude of enrichment were markedly lower than those observed in the FFN and AFCO_2_L groups. This indicates that mechanical stimulation alone can induce metabolic responses, but with less extensive and less pronounced effects compared with interventions that also include a thermal component.

Further validation using spatial transcriptomics data from a vitiligo mouse model subjected to mild heat stress revealed partially overlapping metabolic responses with those observed after FFN and AFCO_2_L treatment. These were primarily characterized by downregulation of lipid, fatty acid, and triglyceride metabolism, accompanied by activation of nucleic acid and carbohydrate metabolic processes. Regarding protein metabolism, the heat-stress group exhibited a downregulation trend solely in the collagen catabolic process, lacking the widespread activation of protein and collagen metabolism observed in the FFN and AFCO_2_L groups. This discrepancy suggests that thermal stimulation alone can induce metabolic reprogramming; however, in the absence of substantial mechanical tissue disruption, its capacity to activate protein synthesis and matrix remodeling programs associated with wound repair remains constrained (Fig. S3).

### Effects of FFN, AFCO_2_L, and FN on immune responses

**Fig. 5:**
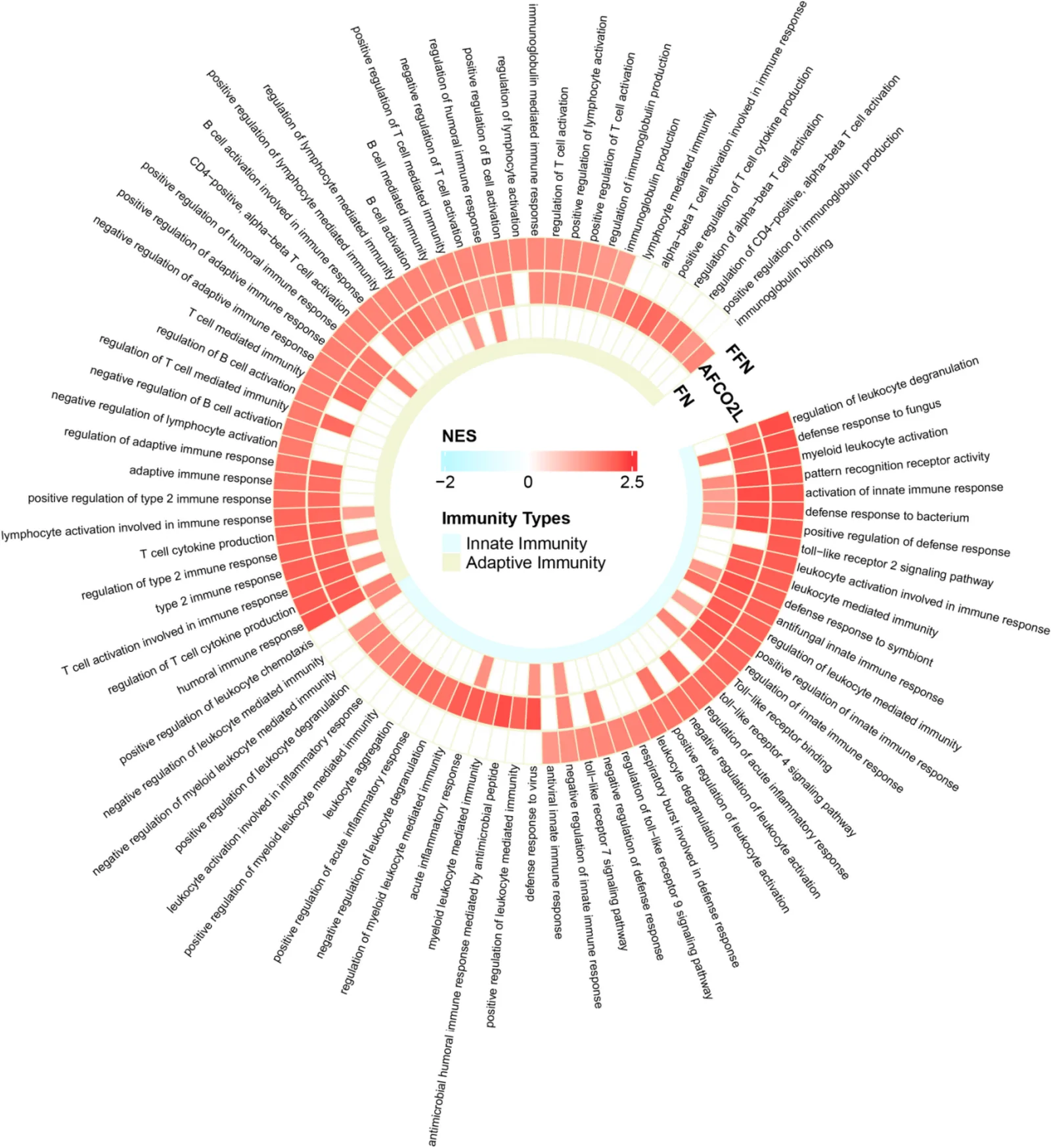
Effects of FFN, AFCO_2_L, and FN on immune responses in skin. Circular heatmap showing immune-related terms from GSEA_GO enrichment results in FFN, AFCO_2_L, and FN groups compared with NC

Within immune-related biological processes, GSEA GO analysis revealed that both FFN and AFCO_2_L induced broad and robust activation of immune responses during the acute phase. Specifically, both groups were significantly enriched in innate immune terms, including leukocyte activation, leukocyte-mediated immunity, defense response to bacterium, and pattern recognition receptor activity. Concurrently, they exhibited enhanced adaptive immune processes such as T cell activation, B cell activation, and humoral immune response. These findings indicate that these two modalities can trigger coordinated activation of multi-layered immune networks at day 1 post-treatment (Fig. 5). In comparison, the FN group also displayed enrichment in immune-related terms; however, both the normalized enrichment scores (NES) and the breadth of enriched terms were substantially lower than those in the FFN and AFCO_2_L groups. Moreover, the immune response in the FN group was predominantly characterized by innate immune activation, with comparatively limited engagement of adaptive immune processes. These findings underscore the distinct intensity and breadth of immune responses induced by the three interventions.

### Disruption of the skin barrier and activation of matrix remodeling and wound repair by FFN, AFCO_2_L, and FN.

**Fig. 6:**
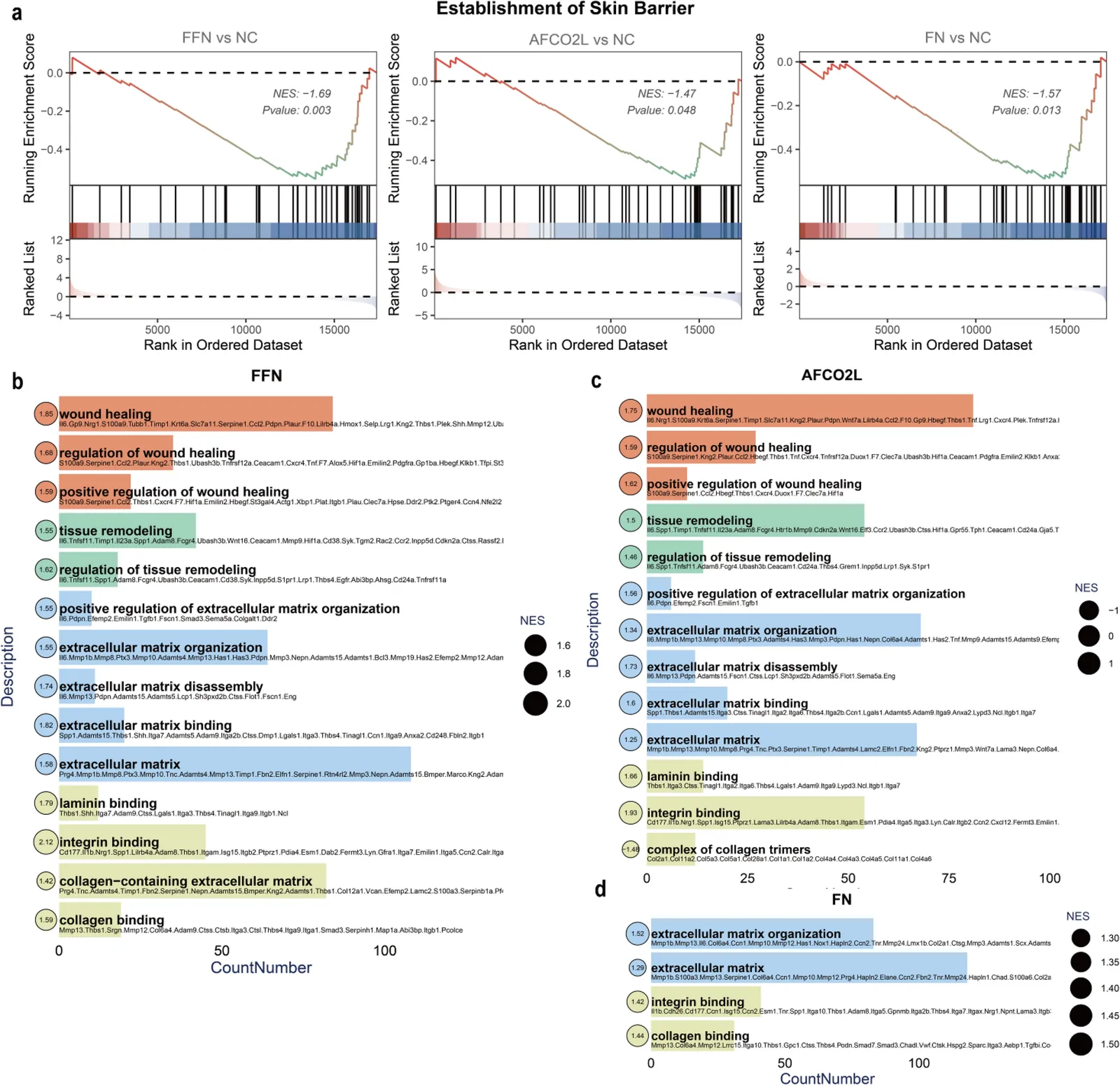
Disruption of the skin barrier and activation of matrix remodeling and wound repair after FFN, AFCO_2_L, and FN treatment. (**a**) GSEA enrichment plots showing the “establishment of skin barrier” term in FFN, AFCO_2_L, and FN groups compared with NC. (**b**–**d**) Bar plots depicting terms related to wound healing and matrix remodeling from GSEA_GO enrichment results in FFN (**b**), AFCO_2_L (**c**), and FN (**d**) groups compared with NC

GSEA_GO analysis demonstrated that the term “establishment of skin barrier” was significantly negatively enriched in all three treatment groups (FFN, AFCO_2_L, and FN) (Fig. 6a), confirming that each micro-injury modality compromised skin barrier integrity at day 1 post-treatment. Concurrently, all three interventions activated matrix remodeling programs associated with skin repair. The FFN and AFCO_2_L groups exhibited highly concordant and significant positive enrichment in wound healing and matrix remodeling-related terms (Fig. 6b, c), including wound healing, regulation of wound healing, positive regulation of wound healing, tissue remodeling, and regulation of tissue remodeling. Furthermore, both groups were significantly enriched in multiple terms related to extracellular matrix remodeling and cell–matrix interactions, such as extracellular matrix organization, extracellular matrix disassembly, collagen-containing extracellular matrix, collagen binding, laminin binding, and integrin binding. These findings suggest that these two interventions can rapidly initiate coordinated repair programs encompassing collagen metabolism, matrix reorganization, and regulation of cell adhesion. In contrast, the FN group exhibited enrichment primarily in collagen metabolic process, extracellular matrix-related terms, and integrin binding (Fig. 6d), with fewer enriched terms overall and lower NES values than those observed in the FFN and AFCO_2_L groups. This indicates that mechanical micro-injury alone can activate matrix-associated repair signals, but with more limited transcriptional depth and scope.

### Effects of FFN, AFCO_2_L, and FN on cutaneous neural, synaptic structure, and function

**Fig. 7:**
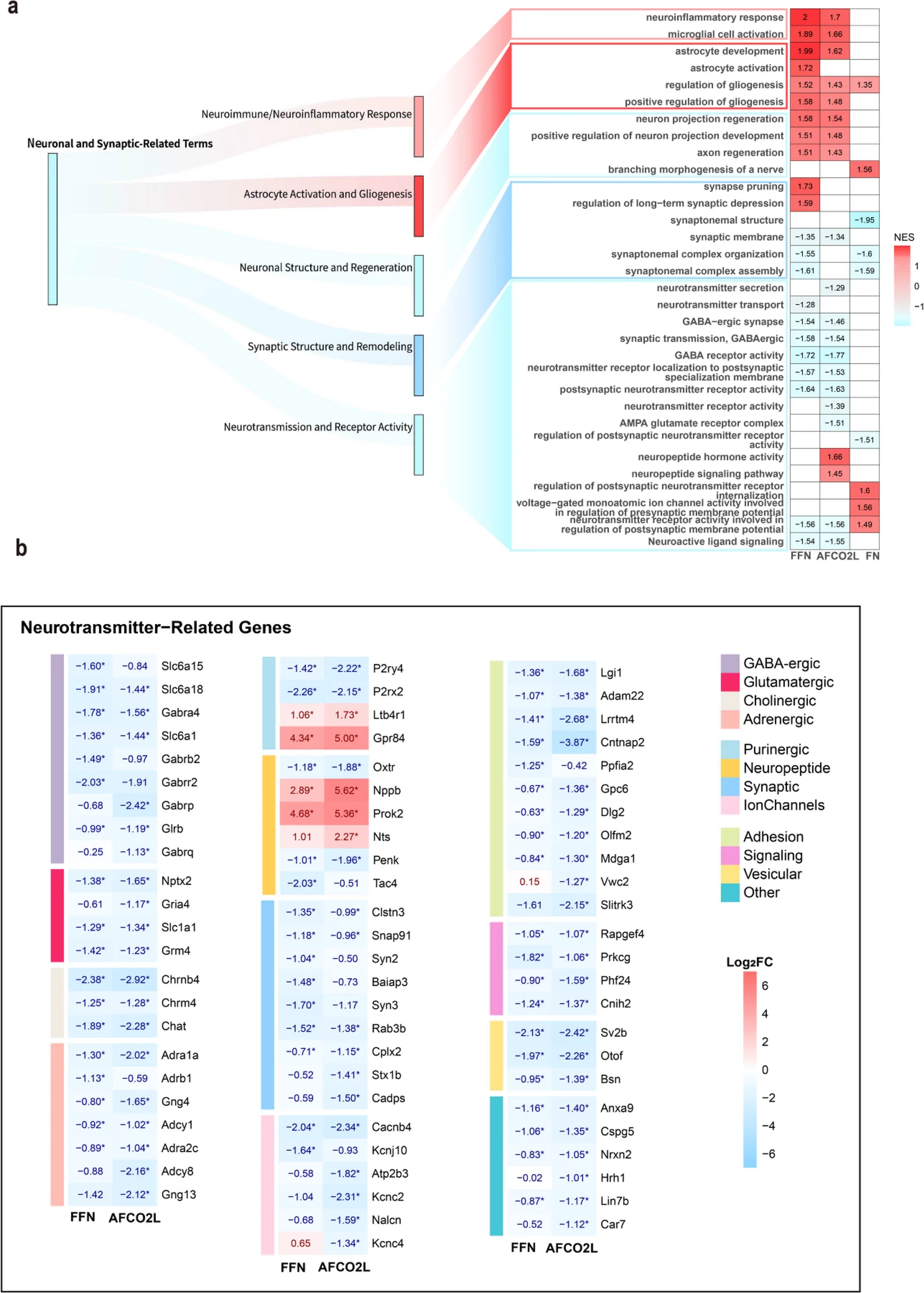
Effects of FFN, AFCO_2_L, and FN on neural, synaptic structure, and function in skin. (**a**) Heatmap displaying neural-related terms from GSEA_GO and GSEA_KEGG enrichment analyses in FFN, AFCO_2_L, and FN groups compared with NC. (**b**) Heatmap showing expression changes of neurotransmitter-related DEGs in FFN and AFCO_2_L groups compared with NC

GSEA_GO and GSEA_KEGG analyses revealed that all three treatments induced both activation and suppression of neural-related transcriptional programs in the skin at day 1 post-treatment. These terms were categorized into five modules (Fig. 7a). Within the neuroimmune/neuroinflammatory response module, both FFN and AFCO_2_L exhibited significant enrichment for neuroinflammatory response and microglial cell activation, with higher enrichment intensity observed in the FFN group. In contrast, no typical neuroinflammatory terms were detected in the FN group. In the astrocyte activation and gliogenesis module, FFN and AFCO_2_L were enriched for astrocyte development, astrocyte activation, and regulation of gliogenesis, whereas FN showed enrichment primarily for regulation of gliogenesis. In the neuronal structure and regeneration module, both FFN and AFCO_2_L were enriched for neuron projection regeneration, axon regeneration, and positive regulation of neuron projection development, whereas FN was mainly associated with branching morphogenesis of a nerve. Within the synaptic structure and remodeling module, FFN specifically exhibited enrichment for synapse pruning and regulation of long-term synaptic depression. Additionally, both FFN and AFCO_2_L displayed negative enrichment for synaptic membrane and synaptonemal complex-related terms, and FN also showed downregulation of synaptonemal complex assembly/structure. Meanwhile, in the neurotransmission and receptor activity module, both FFN and AFCO_2_L consistently exhibited downregulation of neurotransmitter transport and secretion, GABAergic synapse, postsynaptic neurotransmitter receptor activity, and GABA receptor activity. In contrast, the FN group demonstrated a mixed regulatory pattern characterized by receptor internalization, ion channel modulation, and partial changes in receptor activity. Collectively, FFN and AFCO_2_L induced a coordinated neural transcriptional response during the acute phase, typified by neuroinflammation and glial activation, neuronal structural regeneration, synaptic remodeling, and suppression of classical neurotransmitter signaling. In comparison, neural-related transcriptional regulation in the FN group was relatively circumscribed.

To further delineate the specific neurotransmitter systems involved, DEGs associated with neurotransmitter-related terms were systematically analyzed (Fig. 7b). No neurotransmitter-related DEGs meeting the defined thresholds were detected in the FN group. In contrast, both FFN and AFCO_2_L exhibited broader remodeling of multiple neurotransmitter systems at the DEG level, including GABAergic, glutamatergic, cholinergic, adrenergic, and purinergic signaling, accompanied by an overall downregulation of genes related to synaptic vesicle release, ion channels, and downstream signaling pathways. These findings suggest that synaptic transmission and sensory neuron excitability are broadly suppressed during the acute phase. Within this context, certain neuropeptides and purinergic-related molecules displayed relative upregulation, implying a potential shift in neural regulation from high-frequency synaptic transmission toward a lower-frequency, more plastic state.

### Differential transcriptomic responses between FFN and AFCO_2_L

**Fig. 8:**
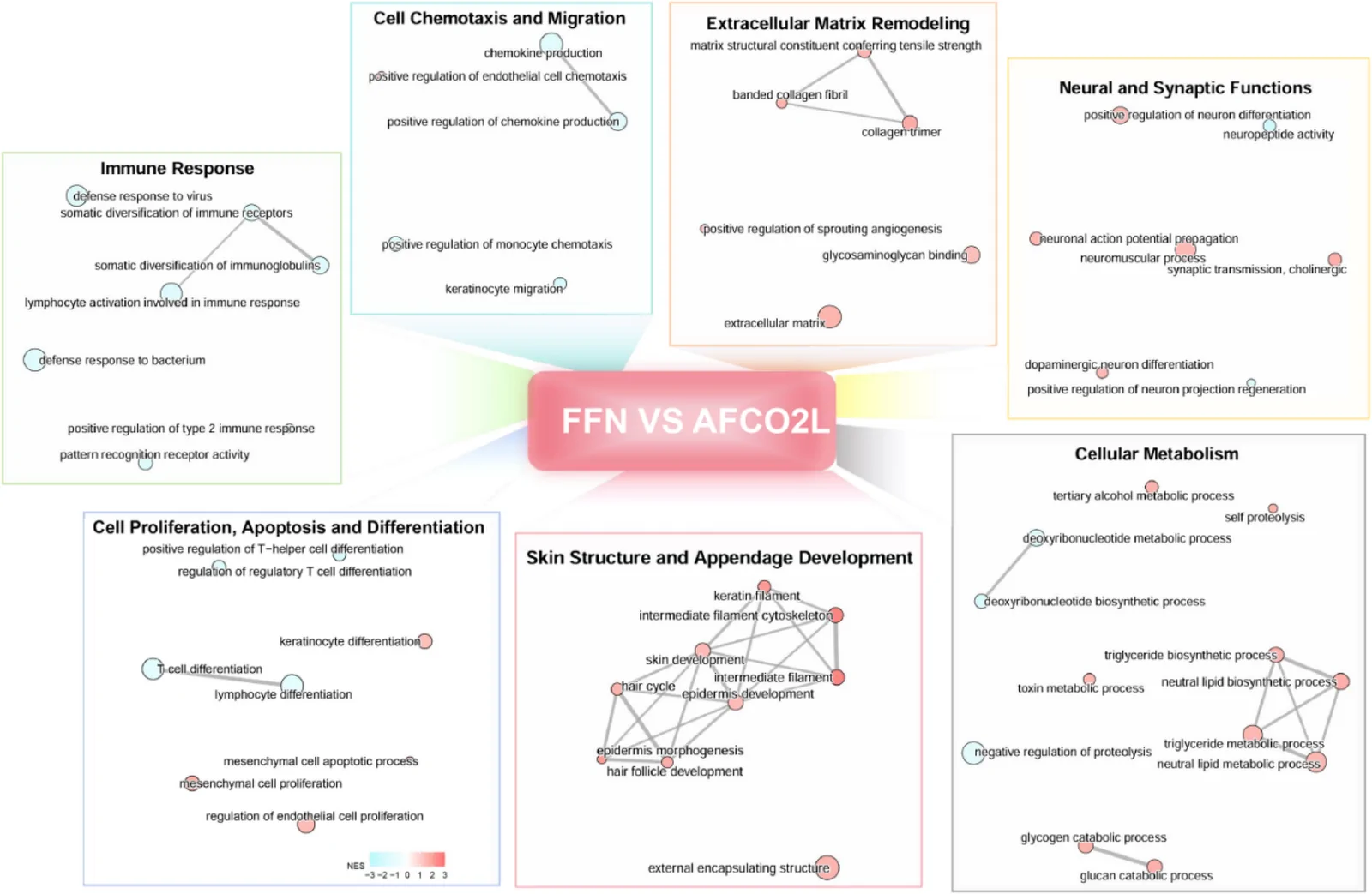
Differential transcriptomic responses between FFN and AFCO_2_L. GSEA_GO enrichment results based on DEGs between FFN and AFCO_2_L groups

Given the highly concordant transcriptional response patterns observed between FFN and AFCO_2_L, we performed GSEA-based GO enrichment analysis on the differential gene expression between these two groups to systematically compare their transcriptomic differences (Fig. 8). Relative to AFCO_2_L, FFN exhibited preferential enrichment in a range of biological processes associated with tissue structure and functional remodeling. These primarily included extracellular matrix remodeling, regulation of neural and synaptic function, skin structure and appendage development (including hair cycle and hair follicle development), cellular metabolism, and tissue-related cell proliferation (endothelial and mesenchymal cells) and differentiation (keratinocytes). Conversely, FFN displayed a relative trend toward negative enrichment in processes related to immune response, immune cell proliferation and differentiation, deoxyribonucleotide metabolism, and cell chemotaxis and migration when compared with AFCO_2_L. Overall, although both FFN and AFCO_2_L induced highly overlapping transcriptional responses during the acute phase, FFN tended to preferentially activate transcriptional programs centered on tissue remodeling, neural regulation, and skin renewal, whereas AFCO_2_L was associated with relatively stronger activation of immune-related and cell migration-related transcriptional features.

## Discussion

In this study, we employed high-throughput RNA sequencing to systematically compare the acute molecular response patterns in mouse skin 24 h after treatment with FFN, AFCO_2_L, and FN. Our analyses revealed both shared and modality-specific transcriptional programs across multiple biological domains. At the level of immune responses, all three modalities significantly activated both innate and adaptive immunity at day 1 post-treatment. This observation is consistent with previous studies of microneedling and ablative fractional laser treatments in human skin, where controlled cutaneous injury was shown to initiate coordinated inflammatory responses and subsequent tissue remodeling processes [8, 13]. Notably, AFCO_2_L exhibited overall stronger enrichment in inflammatory and immune-related terms than FFN and FN. This disparity likely reflects differences in the spatial distribution and temporal dynamics of signaling induced by these micro-injury modalities. The thermal stimulation delivered by FFN is more discrete and transient, whereas AFCO_2_L generates regularly distributed, repetitive microthermal coagulation zones within the skin [14], which may facilitate more coordinated integration and detection of immune-related transcriptional signals at the tissue level.

With respect to cell fate regulation, both FFN and AFCO_2_L coordinately activated transcriptional programs associated with proliferation, differentiation, and apoptosis across multiple cell lineages at day 1 post-treatment, including immune cells, skin structural cells, and neural-related cells. It is important to note that enrichment of astrocyte and microglia-related terms in the transcriptomic analysis does not necessarily imply the actual presence of these central nervous system-specific cell types in the skin. Rather, these signals likely reflect a class of transcriptional states defined by glial-associated functional programs involved in inflammatory regulation and neural support. In peripheral tissues, such programs are typically executed by Schwann cells, perineural supporting cells, and tissue-resident monocyte-macrophage populations. Previous studies have demonstrated that peripheral glial cells can undergo dedifferentiation and re-enter the cell cycle following skin injury. These cells proliferate and migrate into the wound bed, secrete chemokines such as CCL2 to recruit monocyte-derived macrophages, and promote myofibroblast formation and keratinocyte migration through paracrine activation of signaling pathways including TGF-β, thereby contributing to wound contraction and epithelial repair [15–17]. Therefore, the glial-related transcriptional features observed in this study likely represent activation of a network centered on peripheral nerve support cells involved in injury sensing and repair regulation, rather than direct participation of central nervous system-derived cells.

Previous studies of microneedling and fractional laser resurfacing in human skin have demonstrated that these physical stimulation modalities can induce vascular activation and angiogenic responses during tissue remodeling [18, 19]. Additionally, FFN exhibited more pronounced positive enrichment for endothelial cell proliferation and vascular endothelial cell migration-related terms, suggesting a relatively stronger activation of vascular-associated transcriptional programs in the skin. This enhanced vascular response may be attributable to the combined effects of transient high-temperature stimulation and mechanical penetration induced by FFN, which may jointly influence dermal microvascular structures and promote endothelial stress responses and vascular remodeling-related signaling. The vascular responses observed after FFN treatment may provide a mechanistic basis for further investigating its potential application in skin conditions involving impaired vascular function or tissue repair deficits. In comparison, AFCO_2_L promoted differentiation across a broader array of immune cell types, consistent with its more concentrated activation of immune and inflammatory pathways. FN primarily activated a more restricted set of proliferation and differentiation programs, consistent with its predominantly mechanical mode of stimulation, lower degree of tissue disruption, and relatively mild repair response.

At day 1 post-treatment, both FFN and AFCO_2_L markedly activated chemotaxis and migration programs across multiple cell types. AFCO_2_L exhibited enrichment in a greater number of migration-related terms, suggesting stronger activation of migration-associated signaling. Melanocyte chemotaxis and migration are fundamental biological processes underlying repigmentation in vitiligo lesions. Both FFN and AFCO_2_L upregulated the melanocyte migration-related gene Cxcl12, yet diverged in receptor engagement: FFN predominantly enhanced the Cxcl12-Ackr3 (CXCR7) axis, whereas AFCO_2_L preferentially activated Cxcl12-Cxcr4 signaling. Prior studies have shown that CXCR7 mediates directional migration of mature human epidermal melanocytes in response to CXCL12 [20], while the CXCL12–CXCR4 axis guides melanocyte stem cells and progenitor cells within hair follicles to migrate toward the dermal papilla or hair matrix, where they differentiate into mature melanocytes to sustain hair pigmentation. Moreover, during skin injury and repair, dermal fibroblasts and vascular endothelial cells can continuously secrete CXCL12, establishing a chemotactic gradient at the wound edge that promotes migration of multiple CXCL12-responsive cell types [21, 22]. These findings suggest that AFCO_2_L may facilitate repigmentation of vitiligo lesions by promoting the migration of hair follicle melanocyte stem and progenitor cells toward the epidermis via the CXCL12-CXCR4 axis, although this possibility warrants further experimental validation. Notably, both FFN and AFCO_2_L ultimately activated downstream ERK/MAPK signaling in association with Cxcl12-related pathways, indicating that despite differential receptor usage, the two modalities may converge at the level of migration execution. Furthermore, in a vitiligo mouse model subjected to thermal stimulation without overt tissue injury, activation of the Cxcl12-Ackr3 axis was also observed. This suggests that the pro-migratory effects of FFN and AFCO_2_L are not solely contingent upon the classical cascade of inflammation and chemotaxis following micro-injury; thermal stimulation per se may directly contribute to the regulation of melanocyte migration, providing additional mechanistic insight into heat-based therapeutic strategies for vitiligo.

Both FFN and AFCO_2_L have been shown to significantly improve pruritus scores and quality of life in patients with chronic pruritic dermatoses such as neurodermatitis and prurigo nodularis [9, 23, 24]. Previous investigations indicate that chronic pruritus is not merely driven by inflammation but is more intimately linked to persistent hyperexcitability of sensory neurons and alterations in synaptic connectivity and neurotransmitter regulation. A hallmark feature is the amplification and sustained transmission of itch signals along peripheral sensory pathways [25, 26]. In this study, both FFN and AFCO_2_L induced neuroimmune responses, glial activation, and neural structural remodeling at day 1 post-treatment, accompanied by broad downregulation of multiple classical neurotransmitter systems. These findings suggest that these modalities may recalibrate the neuroimmune microenvironment, potentially dismantling aberrant synaptic structures and suppressing synaptic transmission and neuronal excitability, thereby attenuating itch signal propagation. In comparison, FN exerted relatively modest regulation of neural-related transcriptional programs, indicating a more limited impact on neural structure and function, and may therefore be better suited for modulating mild to moderate skin conditions associated with neural hypersensitivity.

In summary, this study provides a systematic transcriptomic comparison of the acute biological effects of three micro-injury modalities on normal skin. All three treatments elicited multi-layered transcriptional responses associated with cell proliferation, differentiation and apoptosis, chemotaxis and migration, immune responses, metabolic remodeling, disruption and repair of the skin barrier, and regulation of neural and synaptic function. This indicates that micro-injury can broadly activate stress and repair programs in cutaneous tissue. Compared with the purely mechanical stimulus FN, FFN and AFCO_2_L provoked broader and more pronounced transcriptional changes across multiple biological processes, particularly in tissue remodeling, immune cell recruitment, metabolic regulation, and neural-related pathways. These findings underscore that thermal stress-associated signaling may amplify injury-induced repair and reconstruction programs, thereby conferring distinct biological effects relative to mechanical injury alone. Notably, several repair-associated transcriptional signatures identified in our mouse model are consistent with previous observations from human studies of microneedling and ablative laser-based skin remodeling, suggesting potential relevance to clinically observed skin remodeling responses.

Nevertheless, several limitations should be acknowledged. First, this study was performed primarily in normal mouse skin and focused on early transcriptomic responses following different micro-injury modalities. Although early activation of extracellular matrix remodeling-related transcriptional programs was observed, later-stage functional outcomes, such as collagen deposition and long-term matrix remodeling, were not directly assessed. Therefore, future studies incorporating additional time points will be necessary to characterize the dynamic progression from early molecular responses to subsequent tissue remodeling events. Second, species differences in skin architecture, hair follicle characteristics, and wound-healing dynamics should be considered when extrapolating findings from mouse models to human skin. Validation in human skin models and disease-relevant systems, together with single-cell or spatial transcriptomic approaches and functional experiments, will help further define the cellular mechanisms underlying skin remodeling and clarify the therapeutic potential of different micro-injury approaches.

## Supplementary Information

Below is the link to the electronic supplementary material.

Supplementary Material 1

## Data Availability

All supporting sequencing data generated in this study can be obtained from the corresponding author upon reasonable request.
