# Supplementary material for: Transcriptomic responses of skin to different micro-injury modalities: fire filiform needling, ablative fractional CO2 laser, and filiform needling
Source: Lasers Med Sci. 2026 Aug 25;41(1):203. doi: 10.1007/s10103-026-05004-1 (PMC13506515; doi:10.1007/s10103-026-05004-1)
Supplement: Supplementary file 1 [file 10103_2026_5004_MOESM1_ESM.docx]

**Figure S1.** Quality control and global transcriptomic features of mouse skin samples at day 1 post-treatment. (a) Box plots showing log_2_-transformed gene expression distributions in NC, FN, FFN, and AFCO_2_L groups. (b) Heatmap showing overall gene expression patterns across the four groups.

**Figure S2.** Expression characteristics of melanocyte migration-related genes and downstream signaling pathways at day 1 post-treatment. (a) Heatmap showing expression levels of melanocyte migration–related genes in NC, FN, FFN, and AFCO_2_L groups. (b) GSEA enrichment plot showing co-enrichment of Cxcl12 and Cxcr4 in ERK1/2 cascade-related terms in the AFCO_2_L group.

**Figure S3.** Metabolism-related terms from GSEA_GO enrichment results comparing the heat and control groups based on spatial transcriptomics data from a vitiligo mouse model.
